# Is legal status impacting outcomes of group therapy for posttraumatic stress disorder with male asylum seekers and refugees from Iran and Afghanistan?

**DOI:** 10.1186/1471-244X-13-148

**Published:** 2013-05-24

**Authors:** Boris Drožđek, Astrid M Kamperman, Wietse A Tol, Jeroen W Knipscheer, Rolf J Kleber

**Affiliations:** 1 Psychotrauma Centrum Zuid Nederland/Reinier van Arkel groep, Den Bosch, The Netherlands; 2 Department of Psychiatry, Erasmus MC, Rotterdam, The Netherlands; 3 Department of Mental Health, Johns Hopkins Bloomberg School of Public Health, Baltimore, USA; 4 Department of Clinical and Health Psychology, Utrecht University, Utrecht/Arq Foundation, Diemen, The Netherlands

**Keywords:** Post-traumatic stress disorder, Asylum seekers, Refugees, Group therapy, Resettlement stress, Iran, Afghanistan

## Abstract

**Background:**

Legal status and other resettlement stressors are known to impact mental health of asylum seekers and refugees. However, the ways in which they interact with treatment of posttraumatic stress disorder (PTSD) with these populations is still poorly understood. The aim of this study was to examine whether legal status and other resettlement stressors influence outcomes of a trauma-focused group PTSD treatment within a day-treatment setting with asylum seekers and refugees.

**Methods:**

Sixty six male Iranian and Afghan patients with PTSD residing in the Netherlands were assessed with self-rated symptom checklists for PTSD, anxiety and depression, and a demographic questionnaire one week before and two weeks after the treatment. Multivariate linear regression analysis was used to examine the impact of legal status and living arrangements on the treatment outcomes per symptom domain.

**Results:**

The results suggest that both asylum seekers and refugees can be helped with their mental health complaints with a trauma-focused group therapy for PTSD regardless of their legal status. Obtaining a refugee status in a course of the treatment appears to improve the treatment outcomes.

**Conclusions:**

Legal status is impacting outcomes of group therapy for PTSD with male asylum seekers and refugees. Asylum seekers may benefit from group treatment regardless of unstable living conditions.

## Background

According to the United Nations High Commissioner for Refugees [1], there were 10,4 million refugees, 15,6 million internally displaced persons, and 6,6 million stateless persons worldwide in 2009. Those arriving in host areas such as Europe, North America and Australia submit a request for asylum and are, therefore, considered as asylum seekers. Some of them are immediately rejected, and either repatriated or remain illegally in a host country. Others may have their claims for political asylum rejected, but are temporarily accepted on “humanitarian” grounds because of having an illness such as a posttraumatic stress disorder (PTSD) that can not be treated in the home country. In cases of “humanitarian” visas, a stay in a host country is limited by the length of a medical/psychological treatment. A third group of asylum seekers enters a long lasting legal procedure of seeking a permanent refugee status on political grounds, and for many years, their status can be pending. In all cases, asylum seekers have minimal legal rights and restricted opportunities to participate in a host society. Their situation changes only when they obtain a permanent legal status and become recognized as refugees.

A range of studies has assessed mental health in refugees and asylum seekers (for a review see [2]). Prevalence rates of PTSD in asylum seekers and refugees vary across studies, with the unadjusted rate of 30,6% reported in a large meta-analysis study [3].

Research in community samples [3-5] has shown that both pre-migratory stressors and post-migration/resettlement adversities have a strong impact on mental health of asylum seekers and refugees. Several studies [6-9] focused specifically on the impact of a legal procedure of seeking asylum on mental health, and have suggested that a long-lasting procedure of seeking asylum causes substantial post-migration stress and may lead to retraumatization in exile. Moreover, an earlier Dutch study with Iraqi asylum seekers [10] showed that a long lasting asylum procedure leads to cumulated post migration stress factors. Legal status may play a crucial role in the lives of forced migrants, as it determines presence of many other resettlement stressors. Unlike refugees, asylum seekers are threatened with repatriation on a daily basis, have no legal rights in a host country, are socially and economically marginalized, are forced to live in collective reception centres, and have no rights to reunite with family members left behind.

The relationship between social support and PTSD has also been studied. While some studies [11,12] have found that social support in the aftermath of a traumatic event is a major protective factor against development of PTSD, more recent studies with asylum seekers and refugees [13] suggest a more nuanced relationship. It is assumed that poor social support acts as a risk factor for greater PTSD symptom severity in earlier stages of coping with trauma (e.g., 6–12 months post-trauma), while during later stages of coping (18–24 months post-trauma), greater PTSD severity contributes to an erosion of social support resources [14].

With regard to other resettlement stressors, a meta-analysis by Porter and Haslam [2] indicated that worse mental health outcomes in asylum seekers and refugees are observed for those confronted with more resettlement adversities, like living in collective accommodation, experiencing restricted economic opportunities, and for those who fled from armed conflicts that kept continuing. Other studies with refugees [10,15] suggested that resettlement stressors are important for explaining common mental disorders, while PTSD symptoms are particularly influenced by exposure to torture trauma.

Despite demonstrated poor mental health outcomes in asylum seekers and refugees, there are few studies evaluating mental health services for these populations [16,17]. Studies evaluating group therapy for PTSD in particular are even more scarce. A school-based group psychotherapy program for war-exposed adolescents in Bosnia-Herzegovina [18] was shown to be effective in a randomized controlled trial. Recent controlled cohort studies [19,20] of a trauma-focused group treatment within a day-treatment setting suggested its effectiveness among asylum seekers and refugees in an industrialized setting.

Previously, it has been reported [21] that outcomes of a trauma-focused group treatment for PTSD did not appear to be impacted either by socio-demographic characteristics of asylum seeking and refugee patients or by resettlement adversities, like legal status, living conditions, family ties and major losses or life changes in a course of the treatment. However, that study was of an exploratory nature and performed on a sample that was relatively small and heterogeneous in terms of ethnicity and gender. Also, results of that study suggested that resettlement stressors tend to cluster strongly within populations of asylum seekers and refugees, and that possible effects of socio-demographic variables and resettlement stressors seem to be confounded by their legal status. Therefore, the impact of individual resettlement stressors was difficult to demonstrate.

The main aim of this study was to test whether legal status influences the outcomes of a trauma-focused day-treatment group approach in a homogeneous sample of Iranian and Afghan male help-seeking asylum seekers and refugees with PTSD. Furthermore, we wanted to explore whether living arrangements have a potential additional impact on the treatment outcomes. We expected that asylum seeking patients will benefit less from the treatment than refugees, and that asylum seekers who resettled alone will benefit the least from the treatment.

## Methods

### Design and participants

Male, Farsi/Dari speaking patients from Iran and Afghanistan participated in the study. They were all asylum seekers and refugees in the Netherlands.

The patients were referred by general practitioners for PTSD group treatment to an outpatient facility specialized in treatment of survivors of war, torture, and political violence. Upon referral, they were assessed with a set of screening instruments for PTSD and comorbid psychopathology and clinical interviews aiming at establishing their suitability for participation in the group treatment. The treatment inclusion criteria matched those for group psychotherapy in general [22], but were less exclusive since no prior experiences with psychotherapy and no stable living arrangements were required. For details considering the group treatment inclusion criteria see [20,22]. Patients who were diagnosed with chronic PTSD according to the DSM-IV [23] and have met the group treatment inclusion criteria form the sample of this study. The sample largely corresponds with the sample of an earlier controlled cohort study of this group treatment approach [20].

The group treatment consisted of group psychotherapy sessions combined with non-verbal therapy sessions (psychomotor therapy, art therapy, music therapy). The treatment groups were homogeneous in terms of gender and common language, but varied in (a) the number of days per week in which the program was implemented, and (b) the number of non-verbal therapies applied [20]. Three different forms of group treatment were included in the study: the 3-in-3 group (three non-verbal therapy sessions and two group psychotherapy sessions in 3 days a week), the 3-in-2 group (three non-verbal therapy sessions and two group psychotherapy sessions in 2 days a week), and the 2-in-2 group (two non-verbal therapy sessions (art therapy excluded) and two group psychotherapy sessions in 2 days a week). All groups were facilitated by the same pair of therapists, and followed a treatment manual [22] to ensure consistency of implementation. The treatment was phase-oriented. It combined trauma-focused, empowerment, and supportive interventions (among others advocacy), and was designed to help asylum seekers and refugees work through their traumatic experiences and place them in a life-span developmental perspective. Such a comprehensive approach that acknowledges persons in context and adopts a developmental, life-span perspective in treatment of complex PTSD was also recommended by others [13,24,25]. For details considering the group treatment approach see [22]. The treatment lasted for 1 year. Every calendar year, one treatment group consisting of 8 to 10 patients was run. None of the patients had been treated for PTSD before.

The patients were assigned to a treatment group available at a particular moment within the center’s day treatment program. Treatment assignment was not pre-planned, and the study was part of ongoing monitoring of treatment outcomes at the treatment facility. Data were collected for 7 treatment groups executed throughout 12 years (2000–2011).

### Measures

Resettlement stressors were assessed with a semi-structured questionnaire. In addition to basic demographic characteristics (age, sex, ethnicity, marital status, country of origin), the following resettlement stressors were assessed: legal status (asylum seeker/refugee); living arrangements (living alone/living with family/living as an incomplete family); housing (living in an asylum seeker reception center/common housing); family left behind (spouse/children/parents/other relatives); work (income through paid job/welfare); and fluency in the Dutch language (yes/no). Additional questions were asked on major losses and life changes related to family life, legal status and living arrangements occurring in a course of the treatment.

The Farsi/Dari version [26] of the Harvard Trauma Questionnaire (HTQ) [27,28] was administered in order to inquire about exposure to a variety of types of trauma events experienced, as well as PTSD symptoms according to the DSM-III-R [29]. The trauma events section describing a range of types of traumatic experiences common to asylum seekers and refugees has demonstrated high test-retest reliability (r = 0.89), and the trauma-related symptoms scale demonstrated adequate levels of interrater (κ = 0.93), test-retest (1 week: r = 0.89), and internal (Cronbach alpha =0.96) reliability in Southeast Asian populations. Scores above a cut-off of 2.5 predicted PTSD. The Farsi/Dari translation (Cronbach alpha = 0.85) has demonstrated high internal consistency [26].

To assess psychiatric symptoms commonly co-morbid with PTSD symptoms, that is to say anxiety and depression, the Farsi/Dari versions [26] of the Hopkins Symptoms Checklist (HSCL-25) [30,31] was used. The instrument includes 10 anxiety items and 15 depression items scored on a four-point ordinal severity scale. Scores above a cut-off of 1.75 indicated clinically significant distress. Internal consistency of the Farsi/Dari translation (Cronbach alphas: anxiety = 0.86, depression = 0.85) was found to be high [26].

Both instruments have been validated against clinical diagnoses and widely used across cultural settings [32].

### Procedures

All participants completed the questionnaires one week before the treatment and two weeks after the treatment. At the second measurement, additional questions regarding changes in resettlement stressors occurred in a course of the treatment were added. The questionnaires were completed by participants themselves. Those who were illiterate were helped by a trained professional interpreter. The same interpreter was used for both the screening interviews and the treatment.

Informed consent was obtained upon explanation of the study’s goals and procedures with help of a professional interpreter, and anonymity of responses was guaranteed. Research procedures were consistent with the Declaration of Helsinki and have been approved by the Board of Directors of the first author’s institution (Reinier van Arkel groep,’ s-Hertogenbosch, The Netherlands).

### Statistical analyses

First, the respondents were divided over three subsamples related to their legal status: (1) participants with a permanent refugee status during a complete course of the treatment (N = 12); (2) participants without a permanent status (temporary and pending asylum seekers) who were not granted a status in a course of the treatment (N = 38); (3) participants who were granted a permanent refugee status in a course of the treatment (N = 16). Differences in sociodemographic characteristics, resettlement stressors, treatment characteristics and treatment outcomes between these three subsamples were tested using ANOVA’s for continuous variables and Chi-square tests for categorical variables. Within each subsample we tested the treatment effect using paired T-tests. In addition, Cohen *d*’s and *r*’s [33] were calculated. A Cohen *d* of 0.2 to 0.3 is seen as a small effect; around 0.5 as a medium effect; and 0.8 and higher as a large effect. Pearson *r*[33] can vary in magnitude from −1 to 1, with −1 indicating a perfect negative linear relation, 1 indicating a perfect positive linear relation, and 0 indicating no linear relation between two variables. Effect sizes are interpreted as follows: small, *r* = 0.1 to 0.23; medium, *r* = 0.24 to 0.36; large, *r* = 0.37 or larger [33].

Second, we performed multivariate linear regression analysis using the Generalized Linear Models Module to examine the effect of (changes in) legal status during intervention on the treatment outcomes per symptom domain. The post-treatment symptom level was used as an outcome variable, while the symptom level at baseline was included as covariate. To account for possible differences in the treatment outcomes, the type of treatment was included as a factor variable.

Third, the variable living arrangements (e.g. single/divorced men living alone, married men living alone, married men living with their spouses and a family) was added to the regression analysis.

We performed sensitivity analyses in which we repeated the analyses. For this purpose we combined our subsamples into different categories: (1) participants whose legal status was unchanged (N = 50) versus participants whose legal status changed in a course of the treatment (N = 16), (2) participants without a permanent status (temporary and pending asylum seekers) (N = 54) versus participants with a permanent refugee status at the start of the treatment (N = 12), and (3) participant living with family (N = 34) versus participants living alone (N = 32).

Dummy variables were constructed to analyze impact of the categorical variables. All data were checked for normality. Relationships between variables were checked for linearity (between predictor and outcome variables) and multicollinearity (between predictor variables). Model fit was evaluated using McFadden pseudo R^2^, deviance and Akaike’s information Criterion (AIC). All model fit indices were interpreted as a smaller value indicating a better fit. McFadden R^2^ and deviance compare the final model (with all predictors) to a fully saturated model. A non-significant test statistic means rejection of the null hypothesis that the model does not fit the data, i.e. a non-significant value suggests that the final model fits the data. AIC is a relative goodness of fit measure that allows for correction of complexity of the model. Data on (change of) legal status and living arrangements were cross-validated using information from the additional questions. We dealt with inconsistent data by consulting the patient’s file and/or the patient. Cases with missing variables were excluded from the analysis. We used PASW 17.0 [34] statistical software to perform analyses.

## Results

### Demographics, trauma history and resettlement variables

The sample consisted of 66 male patients, 47 (71%) from Iran, and 19 (29%) from Afghanistan. Their age ranged from 22 to 58 years, with a mean of 38.3 years (SD = 8.2). Forty patients (61%) were married, and 26 patients (39%) were unmarried or divorced. Thirteen patients (19%) claimed to have sufficient mastery of the Dutch language. Two patients (3%) received income through a paid job (one of them lost this job during treatment). All other patients received a form of minimal state welfare (97%). Forty patients (61%) were living in asylum seekers reception centers. All patients (100%) were survivors of torture, and 34 patients (52%) had experienced additional war-related trauma. The number of traumatic events experienced, as assessed with the HTQ [27,28] ranged from 6 to 20, with a mean number of traumatic experiences of 14.7 (SD = 3.0).

At baseline, twelve patients (18%) had a permanent refugee status in The Netherlands. Fifty four patients (82%) were asylum seekers, and had no permanent status. Sixteen asylum seeking patients (24%) obtained a permanent refugee status in a course of the treatment. We examined differences on resettlement variables between subsamples based on the patients’ legal status. Refugees were more often married than asylum seekers, and asylum seekers granted a refugee status during the treatment (Chi2(66;2) = 5.988). Also, asylum seekers were more often living in a reception center (Chi2(66;2) = 19.215). Asylum seekers who were not granted a refugee status had less often experienced both torture and war-related traumas (Chi2(66;2) = 7.828) (see Table 1).

**Table 1:** Socio-demographic characteristics, traumatic experiences and resettlement stressors in three groups of Iranian and Afghan patients

	**Refugee status obtained**	**Asylum seeker status unchanged**	**Refugee status unchanged**	**Test**
	**(N = 16)**	**(N = 38)**	**(N = 12)**	
Age (years)				F(63;2) = 2.531
Range	22-54	24-58	33-55	
Mean (sd)	36.75 (8.48)	37.50 (7.95)	43.00 (7.77)	
Country of birth				Chi2(66;2) = 3.790
Iran	11 (68.7%)	30 (78.9%)	6 (50.0%)	
Afghanistan	5 (31.3%)	8 (21.1%)	6 (50.0%)	
Marital Status				Chi2(66;2) = 5.988*
Married	9 (56.2%)	20 (51.4%)	11 (91.7%)	
Divorced/Single	7 (43.8%)	18 (48.6%)	1 (8.3%)	
Living arrangements				Chi2(66;4) = 9.314
Alone (single man)	5 (31.3%)	18 (47.4%)	1 (8.3%)	
Alone (married man)	1 (6.3%)	6 (15.8%)	1 (8.3%)	
With family	10 (62.4%)	14 (36.8%)	10 (83.4%)	
Living in asylum seekers reception center	9 (56.3%)	28 (80.0%)	1 (8.3%)	Chi2(66;2) = 19.215**
Paid job	-	-	2 (16.7%)	
Mastery of Dutch language	3 (18.8%)	6 (17.1%)	4 (33.3%)	Chi2(66;2) = 1.787
Trauma in home country				Chi2(66;2) = 7.828*
Only Torture	5 (31.3%)	24 (63.2%)	3 (25.0%)	
Only War	-	-	-	
Combination	11 (68.7%)	14 (36.8%)	9 (75.0%)	
Number of traumatic experiences (sd)	15.25 (2.49)	14.18 (3.15)	15.50 (2.97)	F(63;2) = 1.277

* p < 0.05.

** p < 0.01.

### Treatment characteristics and outcomes

There were three 3-in-3 groups, two 3-in-2 groups, and two 2-in-2 groups included in the study. Twenty seven patients (41%) participated in the 3-in-3 group, 22 patients (33%) in the 3-in-2 group, and 17 patients (26%) in the 2-in-2 group. Asylum seeking patients who were not granted a legal status in a course of the treatment more often received the 3-in-3 treatment. As a part of the treatment, almost all (63) patients (96%) received psychotropic medication (serotonin selective reuptake inhibitors [SSRIs], mostly Sertraline), anticonvulsants (Topiramate), and sometimes antipsychotics (Quetiapine). The choice of medication prescribed was motivated by the authors’ clinical experience and recent literature [35,36].

In the total sample, mean changes on symptoms over a course of the treatment were -.58 (SD = .60; Cohen’s *d* = 1.12; *r* = .49; T(66) = 7.873; p < 0.001) for symptoms of anxiety (HSCL-25), -.62 (SD = .56; Cohen’s *d* = 1.25; *r* = .53; T(66) = 8.940; p < 0.001) for symptoms of depression (HSCL-25), and -.59 (SD = .50; Cohen’s *d* = 1.20; *r* = .52; T(66) = 9.688; p < 0.001) for symptoms of PTSD (HTQ). Differences between the subsamples were observed regarding reduction of depression symptoms. No differences between the subsamples were observed regarding symptoms at baseline (see Table 2).

**Table 2:** Treatment interventions, symptoms levels, and symptoms reduction in three groups of Iranian and Afghan patients

	**Refugee status obtained**	**Asylum seeker status unchanged**	**Refugee status unchanged**	**Test**
	**(N = 16)**	**(N = 38)**	**(N = 12)**	
Intervention				Chi2(66;4) = 15.106**
3-in-3	2 (12.5%)	23 (60.5%)	2 (16.7%)	
3-in-2	9 (56.3%)	8 (21.1%)	5 (41.7%)	
2-in-2	5 (31.3%)	7 (18.4%)	5 (41.7%)	
Symptom level at baseline				
Anxiety, mean (sd)	3.26 (0.57)	3.22 (0.42)	3.46 (0.43)	F(63;2) = 1.246
Depression, mean (sd)	3.38 (0.36)	3.22 (0.51)	3.42 (0.40)	F(63;2) = 1.198
PTSD, mean (sd)	3.36 (0.28)	3.24 (0.43)	3.50 (0.46)	F(63;2) = 2.010
Symptom level post-treatment				
Anxiety, mean (sd)	2.41 (0.46)	2.69 (0.51)	3.08 (0.67)	F(63;2) = 5.493**
Depression, mean (sd)	2.39 (0.48)	2.76 (0.47)	2.81 (0.66)	F(63;2) = 3.38*
PTSD, mean (sd)	2.52 (0.43)	2.72 (0.48)	3.03 (0.82)	F(63;2) = 2.95
Symptom reduction over treatment				
Anxiety				
mean (sd)	−0.85 (0.74)**	−0.53 (0.49)**	−0.37 (0.65)	F(63;2) = 2.602
Cohen’s *d*	1.64	1.13	0.68	
*R*	0.63	0.49	0.32	
Depression				
mean (sd)	−0.99 (0.62)**	−0.47 (0.48)**	−0.62 (0.53)*	F(63;2) = 5.588**
Cohen’s *d*	2.33	0.94	1.12	
*R*	0.76	0.42	0.49	
PTSD				
mean (sd)	−0.84 (0.49)**	−0.52 (0.43)**	−0.46 (0.61)*	F(63;2) = 3.074
Cohen’s *d*	2.32	1.14	0.71	
*R*	0.76	0.50	0.33	

* p < 0.05.

** p < 0.01.

### Relations between resettlement variables and treatment outcomes

The first step of the analyses revealed that patients who were granted a permanent refugee status during the treatment experienced more symptom reduction compared to the patients whose legal status had not changed (e.g. those who already had a permanent refugee status, and those who kept their pending or temporary status). Upon controlling for possible differences in outcomes of the treatment type, the impact of a legal status change against unchanged legal status as reference category was B = .589; 95% CI = .222–.956; p < .01 for symptoms of anxiety, B = .371; 95% CI = .044–.699; p < .05 for symptoms of depression, and B = .402; 95% CI = .065–.739; p < .05 for symptoms of PTSD.

In the second step, living arrangements were added to the analyses. The predictors in the model fit the data well, explaining 37–46% of the variability of the symptom outcome measures. However, we found no independent effect of living arrangements on treatment outcomes (see Table 3).

**Table 3:** Independent predictors for treatment effect per symptom domain in Iranian and Afghan patients (N = 66)

	**Anxiety**	**Depression**	**PTSD**
	**B (95% CI)**	**B (95% CI)**	**B (95% CI)**
Intercept	.723 (−.261–1.708)	.449 (−.462–1.359)	.009 (−.972–.990)
Symptoms at baseline	.303 (.034–.572)*	.404 (.163–.645)**	.659 (.368–.951)**
Treatment			
3-in-3	.231 (−.102–.563)	.347 (.051–.642)*	.237 (−.073–.547)
3-in-2	.245 (−.68–.558)	.329 (.053–.606)*	.161 (−.125–.446)
2-in-2	0	0	0
Status			
Refugee status obtained	.541 (.174–.908)**	.335 (.008–.661)*	.400 (.059–.740)*
Asylum seeker status unchanged	.189 (−.170–.547)	−.194 (−.514–.127)	.061 (−.270–.393)
Refugee status unchanged	0	0	0
Living arrangements			
Alone (single man)	.199 (−.069–.466)	.171 (−.071–.414)	.005 (−.253–.263)
Alone (married man)	.069 (−.318–.455)	.219 (−.126–.565)	−.051 (−.415–.313)
With family	0	0	0
Model fit			
McFadden’s R2	.37	.46	.45
Deviance^‡^	14.975; df = 58; p = .258	12.007; df = 58; =.207	13.010; df = 58; =.224
AIC	107.405	92.822	98.119

^‡^ Log-likelihood (LL) of the specified model compared to LL of the saturated model.

* p < 0.05.

**p < 0.01.

Finally, we performed sensitivity analyses in which we repeated the analyses. These analyses supported the independent effect of status change on the treatment outcomes (significance levels for main effect of status change varied from p < 0.01 to p < 0.05).

## Discussion

Research [13] suggests that resettlement stressors strongly impact mental health of asylum seekers and refugees. However, their impact on PTSD treatment with these populations is still poorly understood. This study aimed at assessing the impact of legal status and other resettlement stressors on outcomes of the day-treatment trauma-focused group therapy in a sample of male asylum seekers and refugees from Iran and Afghanistan in the Netherlands. The results of an earlier controlled cohort study [20] suggested that this approach is an effective treatment for PTSD in asylum seekers and refugees exposed to torture and war violence.

The sample of this study consisted of highly traumatized patients with a mean number of 14.7 types of traumatic events experienced. Other studies of asylum seekers and refugees [37-39] reported a mean between 7 and 15 types of traumatic events experienced per person. In the studied sample, no statistically significant differences in the treatment outcomes were found between asylum seeking patients (who were not granted a refugee status during treatment) and refugee patients who had obtained a refugee status before entering the treatment. However, we had expected that the treatment would be more effective for refugees than asylum seekers since asylum seekers are exposed to more clustered post migration living problems than refugees, and post migratory stressors are found to be significantly related to worse mental health outcomes [6,10,40]. In interpretation of our results, we speculate that the group setting may have provided all patients with enough holding, safety and support, has enhanced or enlarged their coping skills and enabled them to manage resettlement stressors in a more adequate way.

Moreover, the results suggest that a change of legal status influences outcomes of the applied treatment. Gaining a permanent refugee status in a course of the treatment was found to decrease psychopathology in the patients, and to improve outcomes of the treatment. In our sample, the patients who were granted a permanent refugee status in a course of the treatment showed stronger reductions on all assessed symptom categories, i.e., anxiety, depression, and PTSD symptoms. This finding is understandable since it was already hypothesized that resettlement stress caused by uncertainty about asylum decisions has a negative impact on the mental health of asylum seekers [9], that PTSD in asylum seekers and refugees is shaped by conditions of ongoing threat and insecurity in a country of asylum [41,42], and that a decrease in distress can be observed upon obtaining a secure legal status (e.g., refugee status or residency) [43]. Another possible explanation for this finding is that gaining a refugee status restored feelings of safety and control over life in patients. These variables have been found to be important mediating factors in PTSD and depression [44]. We speculate that upon legal recognition, asylum seekers receive a boost of hope for the future linked to emerging opportunities to rebuild their lives. Further, this momentum may have increased their motivation for the treatment and have lead to more favourable treatment outcomes.

Although decreased, all the post-treatment symptom scores still remained above the cut-off scores for clinical relevancy, suggesting that the treatment of asylum seekers and refugees is possible regardless of a legal status, but that its outcomes are relatively limited.

However, our results also paradoxically indicate that asylum seekers who were granted a refugee status during the treatment had better treatment outcomes than the patients who already had a refugee status at the start of the treatment. We suggest that some time upon obtaining a refugee status patients may not be fully aware of all post-migratory stressors that they will face in the future while trying to rebuild existence in a host society. These potent stressors may again, in a course of time, limit coping resources in already recognized refugees and influence their profiting from the treatment on a longer term.

Earlier studies [11-13] suggested that, in general, the relationship between social support and PTSD is complex. The same applies to the relationship between social support from a spouse and mental health in trauma survivors. A study of Vietnamese refugees in the US [45] suggested that the relationship with one’s spouse may be the most important one for married adults. Social support from a spouse was found to be a significant predictor of increased life satisfaction in male adults, and a significant predictor of reduced depression. On the other hand, a study with Iranian immigrants in Sweden [46] showed a change in family power dynamics occurring after relocation and resulting in relational stress. A study of male US veterans with PTSD [47] showed that their spouses were both a source of interpersonal stress and an interpersonal resource, and that initial levels of perceived support and stressors did not predict the course of chronic PTSD symptoms.

This study suggests that married men who were separated from their spouses and families benefited most from the treatment. A possible explanation for this finding can be found in relationship to their legal status. Legal recognition may lead to increased hope for family reunion in these newly recognized refugees (i.e. refugees in the Netherlands may request residency status for their spouses upon receiving their own refugee status) resulting in an increased motivation for the treatment and more favourable treatment outcomes. Meanwhile, single men in this study could not profit from spouses’ support, and married men living together with their spouse and family may have been confronted with an erosion of social support due to their PTSD, as suggested in an earlier study [14]. Unfortunately, our sample was too small to test this possible interaction effect of legal status change and living arrangements. Future research in a larger sample is needed to support this speculation.

In this study, we focused on a mere presence of a spouse and other family members, and not on perceived and received social support. In an earlier study of torture survivors in Nepal [48], perceived social support was not found to be related to mental distress. However, a study [49] with Sudanese refugees in Australia suggested that perceived social support from family and from others within their own ethnic community significantly improved mental health functioning in refugees. More research is needed in order to examine the relationship between presence/social support from a spouse and mental health in refugees on a short and on longer terms, as this relationship appears to be complex.

There were no dropouts in this study, possibly as a result of the patients’ high motivation for treatment and an adequate selection for this type of trauma treatment. Also, not a single patient was repatriated or relocated by the immigration authorities in a course of the treatment. With regard to the study’s finding that asylum seekers may benefit from PTSD group treatment, their repatriation, relocation or imprisonment leading to cessation of the treatment may impede improvement of their mental health.

### Limitations

The study has several limitations. Our sample consisted of asylum seekers and refugees diagnosed with PTSD. A selection bias may have occurred, because of the group treatment inclusion criteria. Generalization of the results of this study to the general population of asylum seekers and refugees is therefore uncertain.

Because of our relatively small sample size, and strong clustering of resettlement stressors with the patients’ legal status, we chose to study only a limited number of demographic variables and post-migration/resettlement stressors, leaving out variables that may have an important additional impact on outcomes of the group treatment (such as level of education, rural or urban background, age of migration, etc.). Sensitivity analyses supported our findings regarding the effect of a change of legal status and living arrangements. However, our findings need to be interpreted with caution and future larger studies focused specifically on these variables are needed to support them. These studies should include more assessment moments and more detailed information on resettlement stress in a course of the time.

Resettlement stressors were assessed with a semi-structured self-constructed questionnaire not including a Likert scale. Therefore, some of the results may be difficult to compare with those of other studies, and the study missed the opportunity to create more difference in nuance as of its results.

We have conducted this study on the notion of universality of the PTSD concept, although we are aware of a pitfall that this concept may not capture other trauma responses that may exist in the studied sample for cultural or other reasons. The study took place over a 12-years period, during which there may have been uncontrolled differences among participants who were assigned to the treatment groups, representing cohorts at different stages across the 12-years time span.

### Suggestions for future research

As avenues for future research, more knowledge is needed on the contextual determinants of treatment outcomes in refugee populations. Resettlement stressors should be studied at multiple times in order to strengthen causal explanations for their interaction with treatment outcomes. For example, refugees originating from the same country and resettling in countries with different immigration policies may be studied in order to examine differential effects of resettlement stressors more adequately.

There is a need for longitudinal studies combining both qualitative and quantitative methods, and examining both measurement and meaning of asylum seekers’ and refugees’ experiences [50]. Long-term follow-up studies may create more insights on influence of timing of life events (both positive and negative) across the life-span on mental health and on long-term outcomes of PTSD treatment. For example, it would be interesting to learn more about impacts of life events on mental health across the life course and upon participation in PTSD treatment with regard to sustainability of treatment outcomes. Moreover, research on impacts of life events on mental health prior to and upon legal recognition of asylum seekers may provide further insight into the importance of legal recognition of asylum seekers in fostering their mental health resilience. Such studies may be informative with regard to the complex interplay of existential safety due to legal recognition and PTSD treatments and the ways these factors influence mental health of asylum seekers and refugees on the longer term.

## Conclusions

The results of this study show that both asylum seekers and refugees may significantly benefit from a group treatment with regard to PTSD symptoms. Obtaining a refugee status in a course of the treatment appears to improve the outcomes. Furthermore, married men who are living alone also seem to benefit most from the group treatment. Influence of other resettlement stressors was difficult to demonstrate, because of a tendency for legal status and other resettlement stressors to cluster, leaving small sample sizes for sub-group analyses. In conclusion, the results suggest that asylum seekers may benefit from group treatment for PTSD regardless of unstable living conditions.

## Competing interests

The authors declare that they have no competing interests.

## Authors’ contributions

All authors contributed to the conception and design of the study and to the final version of the manuscript. BD carried out the clinical research and drafted the manuscript, AK participated in the design and performed the statistical analysis, WT, JK and RK participated in coordination and helped to draft the final version of the manuscript. All authors read and approved the final manuscript.

## Pre-publication history

The pre-publication history for this paper can be accessed here:

http://www.biomedcentral.com/1471-244X/13/148/prepub
